# Groping around in the dark for adequate COPD management: a qualitative study on experiences in long-term care

**DOI:** 10.1186/s12913-020-05875-2

**Published:** 2020-11-10

**Authors:** Sara Lundell, Ulla-Maija Pesola, André Nyberg, Karin Wadell

**Affiliations:** 1 Department of Community Medicine and Rehabilitation, Physiotherapy, Umeå University, 901 87 Umeå, Sweden; 2 Department of Public Health and Clinical Medicine, Division of Medicine, Umeå University, 901 87 Umeå, Sweden

**Keywords:** Chronic obstructive pulmonary disease, Municipality, Qualitative content analysis, Sweden, Organisation, Healthcare professionals, Municipal healthcare, Home healthcare, Nursing homes

## Abstract

**Background:**

Chronic obstructive pulmonary disease (COPD) is one of the most common and deadliest chronic diseases worldwide. Since COPD is a chronic and progressive disease, treatment is necessary throughout life. For people with COPD who cannot live independently, long-term care facilities are often required. However, knowledge is very limited about aspects of importance for effective COPD management in these settings in accordance with current treatment guidelines.

The aim of this study was to explore aspects of importance in long-term care facilities for providing interventions according to treatment guidelines for people with COPD, from the perspective of healthcare professionals, in an effort to prove novel knowledge that could be used to facilitate implementation of treatment guidelines in these settings.

**Methods:**

A qualitative study was performed in northern Sweden. In Sweden, municipalities are responsible for providing long-term care. Interviews with 36 healthcare professionals (nurses, physiotherapists, occupational therapists and dieticians) in municipal healthcare were conducted and analysed using qualitative content analysis with triangulation by the authors.

**Results:**

The overarching theme that emerged from the analysis was *Groping around in the dark for adequate COPD management*. This represents healthcare professionals’ experiences of working with a complex diagnosis somewhat overlooked in the municipal healthcare, an underdog in the healthcare system. The groping around in the dark theme further represents the healthcare professionals’ lack of COPD-related competence, lack of interprofessional collaboration, and insufficient communication with the county council. The fragile group of people with COPD and their relatives were considered in need of support adapted to their context, but routines and resources for COPD management were limited. This lack of routines and resources also resulted in professionals being pragmatic and adopting short-term solutions without focusing on specific needs related to the diagnosis.

**Conclusions:**

The COPD management in long-term care settings showed several insufficiencies, indicating a large gap between clinical practice and treatment guidelines for COPD. It is crucial to improve COPD management in long-term care settings. Consequently, several actions are needed, such as increasing professional competence, establishing new routines, acknowledging and making COPD a higher priority, as well as adapting treatment guidelines to the context.

## Background

Chronic obstructive pulmonary disease (COPD) is a widespread disease, with symptoms and comorbidities affecting the whole person [1]. It is one of the deadliest diseases worldwide [2]. There is strong evidence for the positive health effects of such non-pharmacological interventions as physical exercise, patient education and smoking cessation. Consequently, such evidence-based interventions are recommended in treatment guidelines for COPD [1, 3]. Since COPD is a chronic and progressive disease [1], people with COPD require treatment throughout life.

If people with COPD or other chronic diseases are no longer able to live independently, they may need long-term care, whether in, e.g. ordinary housings or nursing homes [4]. Internationally, deficiencies have been reported in the provision of interventions, according to treatment guidelines for people with COPD, in long-term care facilities [4–6]. However, there is still limited knowledge about aspects of importance for COPD management in such facilities. To accomplish a successful implementation of treatment guidelines for people with COPD, it is essential to have knowledge about the context in which people with COPD are treated and about the knowledge and attitudes of healthcare professionals who provide interventions [7].

Therefore, this study aims to explore aspects of importance in long-term care facilities for providing interventions according to the treatment guidelines for people with COPD, from the perspective of healthcare professionals.

## Methods

A qualitative study based on interviews with healthcare professionals in municipal healthcare in northern Sweden was conducted. The consolidated criteria for reporting qualitative research (COREQ) guided the report of the study [8].

### Setting

In Sweden, the municipalities are responsible for providing long-term care, which involves the oldest and frailest people in the population. The study was conducted in the two most northern counties in Sweden. In these counties, a majority of the population live in cities along the coast, and three municipalities in the coastal area were included in this study. One municipality had 7000 inhabitants, while the other two had between 70,000 and 80,000 inhabitants. The population density was 5, 11, and 37 habitants per square kilometre, respectively [9]. The municipal healthcare includes people in several forms of housing. “Home healthcare” includes people living in ordinary housing, with or without home care services, who are not able to go to a primary care centre because of physical, social or psychological reasons. “Nursing homes” include people living in private rooms in units with attending staff, and “short-term residential care” includes people on temporary stay in units with attending staff.

“Healthcare professionals” within municipal healthcare includes licensed professionals, such as nurses, physiotherapists, occupational therapists and dieticians. Healthcare provided by physicians is, however, organised through the county councils. “Care staff”, responsible for the daily care of people included in municipal healthcare, can be employed by home care services, at nursing homes, or in short-term residential care. They can be assistant nurses, which requires one-year of upper secondary education, or care assistants with no education requirements.

### Participants and recruitment

In all three municipalities, a purposive sampling was applied. Unit managers provided names of healthcare professionals interested in participating, and contact with the potential participants was established via email. Asking participants if they could think of any additional colleagues who would be interested in participating was a strategy that increased the number of participants. All healthcare professionals who were contacted agreed to participate, and in total 36 healthcare professionals were included in this interview study (Table 1). They worked in home healthcare, nursing homes and short-term residential care, many of them in more than one of these contexts. Physicians were not included in the study, since the healthcare provided by physicians is the responsibility of the county council.

**Table 1:** Characteristics of included healthcare professionals

Healthcare professionals	Value
Sex/gender (n), women/men	33/3
Age (years), mean (SD)	44 (11.5)
Profession (n):	
● Nurse	● 13
● Physiotherapist	● 11
● Occupational therapist	● 10
● Dietician	● 2
Professional experience (years), mean (SD)	13 (8.2)
Worked in present position (years), mean (SD)	8 (7.4)

*Abbreviations*: *n* Numbers, *SD* Standard deviation

### Data collection

The data collection lasted from February 2017 to January 2018. Semi-structured face-to-face interviews were conducted at the healthcare professionals’ workplace by UMP, who had no earlier relationship with the healthcare professionals. The interviews consisted of open-ended questions following a thematic interview guide (Table 2). Follow-up questions and prompts were used when needed. A majority of the questions had been used in a previous study [10]. In three of the interviews, two or three healthcare professionals were interviewed together, for practical reasons and according to the healthcare professionals’ wishes. The interviews lasted between 18 and 68 min (median of 38 min), and were audio-recorded and transcribed verbatim by a professional transcriber.

**Table 2:** Thematic interview guide with examples of questions

Question areas with examples of questions	
Current work processes aimed at people with COPD	
*- What interventions do people with COPD usually receive?*	
Encounters with people with COPD	
*- What do you think is important in encounters with people with COPD?*	
Experiences of rehabilitation and health promotion interventions for people with COPD	
*- What is your view on health promotion interventions to people with COPD?*	
Conditions for providing evidence-based interventions	
*- What conditions do you have for providing evidence-based, health-promotion interventions to people wih COPD?*	
Knowledge about COPD and COPD management	
*- How do you experience the COPD knowledge among the healthcare professionals?*	

*Abbreviation*: *COPD* Chronic obstructive pulmonary disease

### Data analysis

The transcripts were analysed using qualitative content analysis [11]. SL had the main responsibility for the analysis. First, the interviews were listened to, and the transcripts were read to gain a sense of overall meaning [11]. Then the interview text was inductively coded using OpenCode 4.03 [12]. The codes were compared for similarities and differences and were grouped into categories and subcategories. Finally, a theme that ran through all categories was formulated. The analysis moved back and forth between the whole text and parts of the text [11]. Credibility was striven for with triangulation [13] by the authors. AN and KW read a selected sample of the interviews and, repeatedly, throughout the analysis process, discussed subcategories and categories with SL. The authors contributed with various competencies and perspectives.

## Results

A theme formulated during the analysis, *Groping around in the dark for adequate COPD management*, pervades the five categories and 17 subcategories that emerged from the data (Table 3).

**Table 3:** Theme, categories and subcategories

Theme	Groping around in the dark for adequate COPD management				
Category	Complex patient group	Organisational insufficiencies	Barriers in communication	COPD-related support needs	Overlooked diagnosis
*Sub-category*	*A fragile patient group* *Questionable knowledge and motivation* *Adapted disease management* *Relatives - a resource in need of support*	*Lack of resources* *Professions in the dark* *Collaboration when needed*	*Underdog in the healthcare system* *Disconnected medical charts* *Random reports*	*Lack of COPD competence* *Lack of COPD routines* *Need for support in COPD management*	*Rely on low-skilled care staff* *COPD as a side-issue* *Focus on case and problems* *Putting out fires*

*Abbreviation*: *COPD* Chronic obstructive pulmonary disease

### Groping around in the dark for adequate COPD management

The overarching theme represents the healthcare professionals’ experiences with COPD management in municipal healthcare. It highlights a frustration among them, that they were not being able to support people with COPD as desired. They perceived that both people with COPD and their relatives needed support adapted to them and their context. However, organisational shortcomings and a lack of competence and routines in the area of COPD, led to the healthcare professionals groping around in the dark, and COPD diagnoses being left somewhat ignored.

### Complex patient group

This category refers to the view of healthcare professionals with regard to the need for providing support adapted to peoples' fragility, motivation, context, and relationships.

People with COPD were considered by healthcare professionals to be **a fragile patient group**, with both physical and psychological problems. Overall, people in municipal healthcare were described as fragile and old, especially those in nursing homes, and dementia was common. In addition, people with COPD were perceived as isolated in both home healthcare and nursing homes. Treatment plans for this group were rare, unless they had been hospitalised recently. Healthcare professionals perceived an increase in the number of people with COPD enrolled in municipal healthcare. However, they also believed that many died before they reached municipal healthcare, and that others were undiagnosed.

**Questionable knowledge and motivation** was described being prevalent among people with COPD. Disease severity was suggested to influence knowledge level, with knowledge being greater among people with more severe forms of the disease. Varying knowledge and motivation influenced the willingness of people to accept support, and one view was that smoking status provided a clue as to general motivation:

*... if they have stopped smoking, you then know that perhaps they are more motivated to get better; and if they simply just keep on smoking, then you know that there may be some difficulty... Perhaps they don’t really have the motivation they need.*

(Occupational therapist).

After all, education and support for people with COPD were considered important in increasing motivation and facilitating interventions. However, it was difficult to balance between preparing them for future deterioration and providing sufficient information.

**Adapted disease management** was experienced as being necessary for fragile people and their home environments. Assessment tools such as the six-minute walk test were not judged feasible in municipal healthcare. In addition, interventions, such as supporting food intake and finding an adequate level of exercise were considered more complicated in ordinary housing compared to nursing homes:

*People who are seriously ill, they also have to cope with their everyday lives. And then if I, as a physiotherapist, intervene and use up so much of their energy means that they find it difficult to cope with the rest of their life that they have at home.*

(Physiotherapist).

Further, healthcare professionals found it easier to have a holistic view and to take charge of the situation in nursing homes, whereas the encounters in people’s homes made the relationship between healthcare professional and patient more equal.

**Relatives** were seen as **a resource in need of support**, and healthcare professionals needed to consider peoples' social situations. Relatives were mainly considered to be a resource who could support the patients, but the engagement varied and could also be too extensive, thus making the patient passive. The delicate balancing act between being both a relative and a caregiver, made it important for healthcare professionals to provide support. The experience was that relatives needed more knowledge about COPD. Few relatives knew about what support they were entitled to in the municipality, such as contact with a relative consultant or relief from their caregiver role, where the person with COPD could get temporarily support from home care services or go to short-term residential care.

### Organisational insufficiencies

This category highlights an organisation with many loose ends influencing disease management. The healthcare professionals rarely collaborated, which left some professions hidden in the organisation.

A **lack of resources** was highlighted, regarding both personnel, time and equipment. Healthcare professionals expressed a need for more equipment such as spirometers and pulse oximeters to provide high-quality care. A high workload led to limited opportunities for staying updated, developing and using the routines that were available. Resources for health promotion were not prioritised, which was considered contradictory:

*They want it to look nice and that the statistics look good, and we should not send people to the hospital, and we should work with health promotion at home ... in that way, it becomes prioritised. There is a lot of talking, however, that perhaps the resources don’t end up being where they should be.*

(Nurse).

Long distances made it difficult to provide support to rural areas equal to that of urban areas. In addition, high demands were placed on people’s economic situation and social networks when they needed aids outside the standard assortment, which needed to be bought by the patients themselves.

Dieticians and occupational therapists experienced themselves as **professions in the dark**, while nurses and physiotherapists were considered more visible. Dieticians were employed by the county councils, which complicated their work in municipal healthcare, and nurses had the daily responsibility of nutritional issues. Dieticians worked mainly as consultants with the nurses, acting as intermediaries between them and the patients. Nurses suggested that dieticians were important to the COPD care, while at the same time they contacted them only in special cases and sometimes even forgot about them. Occupational therapists experienced that few people in general had knowledge about their profession and working methods, which gave daily living activities and occupational therapy low prioritisation and left them almost forgotten.

Healthcare professionals only described interprofessional **collaboration when needed**, e.g. initiating contacts for questions or to make requests for interventions. Collaboration regarding COPD was considered rare; instead the professionals worked independently, and team meetings were the only routine collaboration they had. A desire for more collaboration to complement each other’s competences was emphasized:

*... and then we go in and we observe how they are doing. And then things can be discussed later ... how did you experience the situation with the patient? What was the patient able to accomplish and what do they need some help with... We come together in our assessment, you might say.*

(Occupational therapist).

**Fig. 1:**
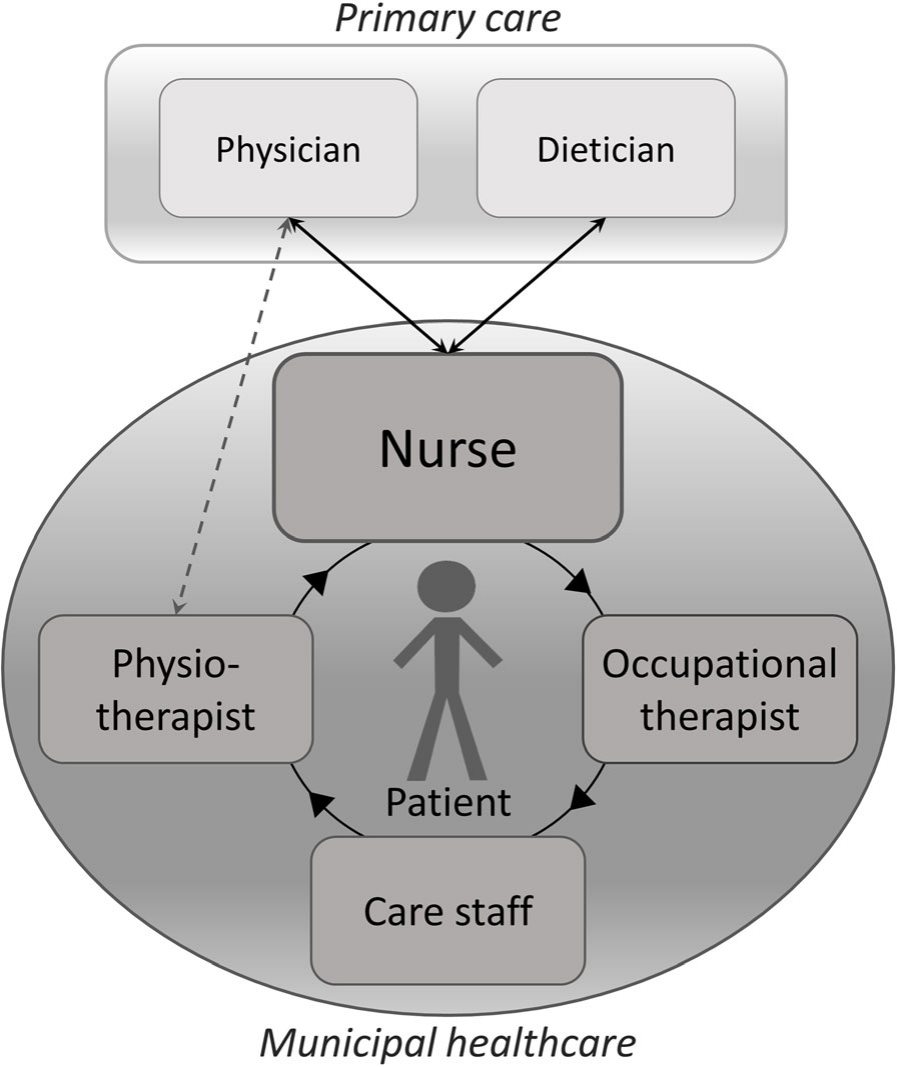
Illustration of described contacts between the patient and professions in municipal healthcare and primary care, with the nurse in a central role in the communication

Nurses had a central role in municipal healthcare, where they had the main responsibility for working with professions in primary care, as well as serving as points of contact between patients and physicians and dieticians (Fig. 1). Care staff was considered the extended arms of healthcare professionals, available to support the patients with interventions. Overall, existing contacts and collaboration was seen as working well, especially when offices were in close proximity and when there was continuity among the personnel.

### Barriers in communication

This category captures how communication across the healthcare system was prevented by unclear responsibilities, separate medical charts and inadequate reports.

Municipal healthcare was perceived as an **underdog in the healthcare system**. The county council was considered the “primary instance” for which treatment guidelines were written. The responsibility of municipal healthcare was considered unclear, and healthcare contacts for people with COPD were unstructured, where some people had regular contact with the county council, while others had contact only within municipal healthcare. A great responsibility was placed on the nurses when physicians only were consultants and no specialised COPD nurses were available in municipal healthcare. Aside from the nurses’ regular meetings with physicians in primary care, they had no routine collaboration with the county council, and it was sometimes experienced to be challenging to receive support from the county council.

Due to **disconnected medical charts**, healthcare professionals experienced limited communication between the municipal healthcare and county council.

*It’s only a one-way communication. I can only see what the county council has written, but they cannot see what I write in our medical charts. This is unfortunate. We are hoping that there will be a change in the future, so that they will be able to read our medical charts and have access to them. But we’re not there yet.*

(Physiotherapist).

The county councils’ lack of access to municipal healthcare medical charts, caused problems especially for the dieticians and physicians employed by the county council. There was fear that this one-way communication would lead to information loss. In addition, healthcare professionals were depending on the county council giving reports with patient information.

However, there were experiences of **random reports** in the contacts with the county council.

*Sometimes, when I am lucky, I get a medical history or epicrisis from the hospital that says a little more. But there’s so very little one gets from the healthcare centre. Rather, quite often one has to do research to find things out on one’s own.*

(Nurse).

Healthcare professionals perceived reaching their colleagues in the county council for a report as a challenge. In addition, the amount, the content and the quality of the reports varied a great deal, with more information from specialty care than from primary care. Written reports were preferred in order to ensure that correct information was received, although several reports were only oral, if they received any report at all.

### COPD-related support needs

This category reflects the healthcare professionals’ insufficient competence and routines with respect to COPD management, in combination with low management support regarding further education.

In general, healthcare professionals perceived a **lack of COPD competence**, which caused insecurity.

*... I feel that I ... in COPD ... I do not feel that I have ... know exactly what to ask for, because I feel I don’t have full knowledge about it. So I know what breathing problems and respiratory complaints can do, and things like that ... but I can’t say I have a full understanding of COPD.*

(Occupational therapist).

Few healthcare professionals thought their COPD-related knowledge was good enough. The broad spectra of diagnoses in the municipal healthcare were seen as a barrier for gaining a deeper knowledge of different diseases. Nevertheless, they expressed having more knowledge of other conditions, such as diabetes. The experience in working with people with COPD varied, but was in general low, and previously gained knowledge of COPD was considered lost as it was not used regularly. Healthcare professionals had a desire to improve or update their COPD-related knowledge about symptoms, diagnostics, assessments, and appropriate interventions.

Healthcare professionals expressed a **lack of COPD routines**, or of having little knowledge about available routines and guidelines. Again, routines for other conditions such as diabetes and stroke were more available and well known.

*Yes diabetes, there we have a bit more knowledge, that an annual follow-up should be done. But with COPD, it doesn’t seem the same. I don’t know if we’re doing any follow-ups with some spirometry or something like that. And I would think that COPD must be equivalent to diabetes as well. So it’s a flaw.*

(Nurse).

In addition, interventions by physiotherapists and occupational therapists were rarely specified in local routines. Consequently, COPD-related interventions became instinctive and dependent on the healthcare professional’s way of working. Implementation of COPD routines in daily work was desired for structure, and to facilitate the provision of equal care.

Healthcare professionals had a **need for support in COPD management**, since they experienced bearing great responsibility in their work. They feared that low knowledge increased the risk of missing routines for COPD.

*... there’s a deficiency there ... because we don’t have training in which annual examinations should be done ... so it might fall between the cracks then. Unless the healthcare centre reminds us and tells us exactly what to do...*

(Nurse).

Further education in COPD, apart from undergraduate studies, was rare, while the healthcare professionals had received further education in other diseases. The municipalities were not perceived as prioritising further education in this area. Instead, it was up to the healthcare professionals to gather knowledge on their own, e.g. by turning to healthcare professionals in the county council, who were believed to have a better track on people with COPD.

### Overlooked diagnosis

This category points to how COPD management was adapted to the context and organisation, leading to emergency solutions where the diagnosis was ignored.

In their daily work, healthcare professionals had to **rely on low-skilled care staff**, since the care staff were considered to have, in general, a low level of COPD-related knowledge. Still, they were seen as “the eyes and ears” of the healthcare professionals, meaning that care staff evaluated the patients’ health status and interventions in order to report problems to the healthcare professionals. However, care staff were found to report problems too late or to have different views on the problems, and their lacking experience with COPD was suggested as a reason for these issues. The level of education among care staff varied, from care assistants in their first job to assistant nurses with an earlier education in care, and healthcare professionals found it important to educate and support them in COPD management.

Healthcare professionals described **COPD as a side-issue** in municipal healthcare. They were not always informed about a COPD diagnosis, as it was seen as *“something that is off to the side sometimes”*. They were unsure of which people had COPD diagnoses, since contact with them was rarely initiated solely on the basis of COPD.

*... then it rarely comes down to COPD being the main diagnosis, it is often some other illness or injury that results in activity limitations. And so COPD becomes like a little... yes actually – a side-issue.*

(Occupational therapist).

They were afraid that, as a consequence, the COPD diagnosis could be missed and that people with COPD were provided low-quality care in the municipality. It was seen as being important to be informed about the COPD diagnosis and to provide more and better healthcare for this patient group by developing local care programs based on national guidelines.

In their work, healthcare professionals had their **focus on case and problems**, meaning that their assessments and interventions focused on and were adjusted to the given case or problem and not to the diagnosis. They found it difficult to reach people to whom they had not been assigned. Knowledge about patients’ diagnoses was sometimes judged as unimportant, and at other times it was judged as an important clue for potential problems, and even crucial when planning rehabilitation and considering potential restrictions in diagnoses such as fractures and stroke. Nurses described themselves relying on the assessments and prescriptions of the physicians rather than on national guidelines.

The focus in municipal healthcare was on **putting out fires** rather than working on long-term solutions. Being informed about the patients at a late stage made it difficult for them to provide satisfactory help to the patients.

*I have experienced so far that I am really not involved when maybe my input would be most useful.*

(Occupational therapist).

Municipal healthcare carried out work based on order of priority. Acute situations influencing patient safety were of the highest priority, while health promotion and preventive interventions were of the lowest priority. Healthcare professionals emphasized that they would like to work more preventively in order to increase the benefits for patients. Still, they experienced that their workload and late involvement forced them to focus on fixing rather than preventing.

## Discussion

To accomplish a successful implementation of treatment guidelines, knowledge about aspects of importance for COPD management at all levels of healthcare is important [7]. Still, few studies have explored aspects of importance for COPD management in long-term care facilities. This is, to our knowledge, the first study to explore aspects of importance for COPD management in Swedish municipal healthcare. The theme “Groping around in the dark for adequate COPD management” illustrates the main results of this study. Insufficiencies in healthcare professionals’ competence, organisation and routines made healthcare professionals struggle with COPD management. The COPD management in municipal healthcare showed several similarities with experiences previously reported in primary care [10], although with more widespread shortcomings in municipal healthcare.

### Results in relation to previous research

#### Lack of COPD competence among healthcare professionals

A need for more competence and support in COPD management was one reason the healthcare professionals were “groping around in the dark”, also reported for palliative care [6]. Thus, nurses instead relied on the judgement of physicians rather than their own, which requires the physicians to have good COPD-specific knowledge. However, lack of COPD-specific knowledge among physicians and other healthcare professionals has been reported earlier [6, 10, 14, 15], and this can be a barrier to adequate COPD management [15, 16]. At the same time, the healthcare professionals felt that they had better knowledge about other diseases and their management than of COPD, which has previously also been reported in Swedish primary care settings [10]. The low prioritisation of further education in long-term care may lead to healthcare professionals not prioritising increased knowledge about low-status diseases such as COPD. A lack of sufficient education in COPD is a challenge in long-term care settings [4], while improved patient outcomes have been shown when nurses in such settings have been specially trained for respiratory diseases [17]. Higginson and Parry [18] suggest that opportunity should be given to increase respiratory competence to improve patient outcomes and decrease the risk for hospital admissions. This suggestion is supported by an official policy statement by the American Thoracic Society and the European Respiratory Society [19]. The responsibility of supporting and increasing the competence of healthcare professionals lies partly on the healthcare professionals themselves, but particularly with the management in health organisations and educational institutions [18, 20].

#### Organisational insufficiencies

The healthcare professionals’ work was also influenced by insufficiencies in the organisation, such as lack of resources, interprofessional collaboration and routines for COPD management. This led to COPD being overlooked, similar to the situation in primary care [10]. Limited resources, which negatively influence the quality of COPD management, have also been previously reported in long-term care settings [4, 21], as well as in other settings [10, 22]. Limited resources have, in general, been reported as a barrier for adherence to treatment guidelines [17]. It is known that the access to interventions according to treatment guidelines for COPD is low internationally [23–25], as well as in Swedish contexts [26–28]. In primary care settings, where most Swedish people with COPD are treated, healthcare professionals experienced that COPD was a low priority [10, 29], which seems to be the case even in long-term care settings. To raise the priority of COPD, COPD must be acknowledged in both long-term care settings and in other settings, which in turn can improve patient outcomes and decrease costs [4]. Changes in a given organisation, e.g. to acknowledging COPD and giving it a higher priority, can be challenging to implement. Nilsen, et al. [30] showed that it is essential to involve the healthcare professionals in the process of change, as they need to understand its necessity and its advantages. To carry off a successful implementation, it is also important that healthcare professionals experience that the organisational change will provide clear benefits for the patients [30].

#### Inadequate communication

Another clear challenge for COPD management in the municipal healthcare was found to be inadequate communication with healthcare professionals in the county council. These communication issues between different levels in the healthcare system have also been highlighted by healthcare professionals in Swedish primary care [10], as well as in other European countries [22]. To provide continuity in care, and high-quality care pathways between healthcare organisations, it is crucial to have well-functioning communication between healthcare professionals [4]. Poor communication might explain the healthcare professionals’ uncertainty regarding which people had a COPD diagnosis. Furthermore, the reported prevalence of COPD in primary care in Sweden was unreasonably low in primary care compared to the national estimate [10], which may indicate a significant structural problem. In addition to the insufficient communication between municipal healthcare and county councils, the collaboration between the professions within the municipal healthcare was also limited. Also in other COPD-related settings in Sweden and Denmark, interprofessional collaboration was experienced as limited, where the organisation and current working methods were presented as barriers [6, 10]. Interprofessional collaboration is highlighted as a crucial part of COPD management in both national and international guidelines [3, 31] and good collaboration can facilitate the implementation of treatment guidelines [17].

### Strengths and limitations

In this study, we have strived to achieve trustworthiness [11] in several ways. First, to achieve *credibility* [11], we included healthcare professionals with different professions, in different municipalities, of different ages, and with varying professional experiences. However, in these settings, most healthcare professionals are women, which is reflected in the healthcare professionals who participated in this study, 92% of whom were women. More male healthcare professionals, as well as the inclusion of physicians and a municipality from more southern parts of Sweden, would have enriched the study. However, since healthcare provided by physicians is not organised through the municipal healthcare in Sweden, physicians were not included in this study. Credibility was also sought by presenting representative quotes in the results section. Second, to achieve *dependability* [11], the question areas presented in Table 2 were used for all interviews, and the interviews in each municipality were performed within a short time period. Finally, to achieve *transferability* [11], we have given a detailed description of the context and methods used, as well as a rich presentation of the results with quotes, guided by the COREQ guidelines [8].

## Conclusions

The findings in this study highlight a considerable gap between treatment guidelines for COPD and the COPD management in municipal healthcare. The healthcare professionals we interviewed experienced organisational limitation and a lack of COPD-related competence and routines, resulting in overlooked COPD diagnoses. Consequently, healthcare professionals in municipal healthcare were *groping around in the dark for adequate COPD management*. To improve the management of people with COPD, the COPD-related competence of healthcare professionals in long-term settings needs to be increased. Furthermore, COPD needs to be acknowledged and given higher priority, and new routines such as interprofessional collaboration are crucial to decrease the gap between COPD management and treatment guidelines for COPD. The interventions recommended in the treatment guidelines should also be adapted to fit the municipal healthcare context.

## Data Availability

The transcripts from the interviews used and analysed during the current study are available from the corresponding author on reasonable request; however, they will only be shared in part (to ensure confidentiality).

## Abbreviations

COPD: Chronic obstructive pulmonary disease
COREQ: The consolidated criteria for reporting qualitative research
n: Numbers
SD: Standard deviation
